# Supplementary material for: A Global Screen for Assembly State Changes of the Mitotic Proteome by SEC-SWATH-MS
Source: Cell Syst. 2020 Feb 26;10(2):133–155.e6. doi: 10.1016/j.cels.2020.01.001 (PMC7042714; doi:10.1016/j.cels.2020.01.001)

P13798 | ACPH_HUMAN | APEH D3F15S2 D3S48E DNF15S2
Monomer MW [kDa]: 81.225 Monomer expected elution fraction: 42

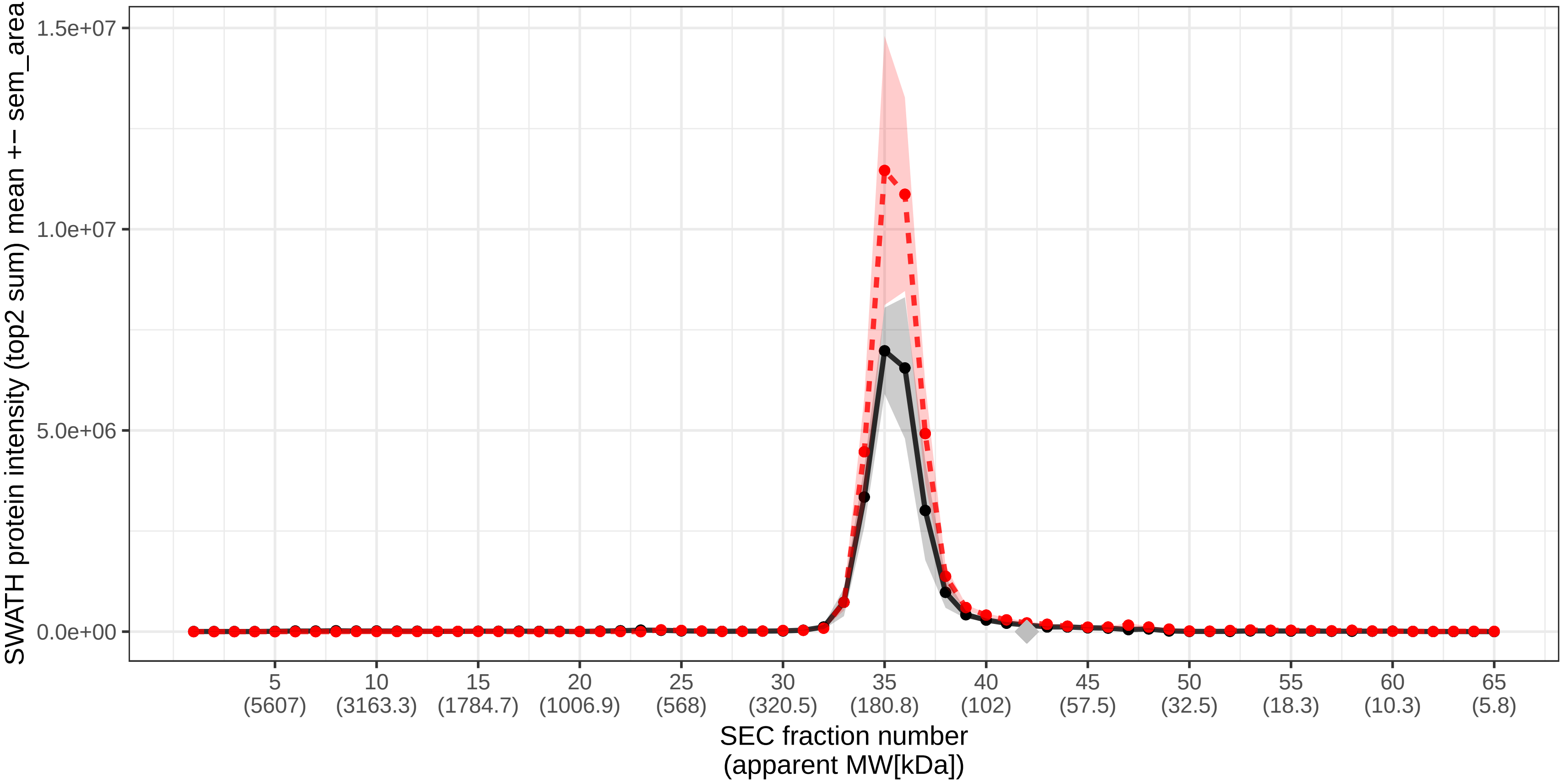

Supplement: Data S1. SEC-SWATH-MS Protein Chromatograms, Related to Figure 1 [file mmc6.zip › SECchrom_P13798_ACPH_HUMAN_APEH_D3F15S2_D3S48E_DNF15S2.pdf]
